# Promising survival for patients with glioblastoma multiforme treated with individualised chemotherapy based on *in vitro* drug sensitivity testing

**DOI:** 10.1038/sj.bjc.6601376

**Published:** 2003-11-11

**Authors:** Y Iwadate, S Fujimoto, H Namba, A Yamaura

**Affiliations:** 1 Department of Neurological Surgery, Graduate School of Medicine, Chiba University, Inohana 1-8-1, Chuo-ku, Chiba 260-8670, Japan; 2 Division of Chemotherapy, Chiba Cancer Center, Nitona 666-2, Chuo-ku, Chiba 260-8717, Japan; 3 Department of Neurosurgery, Hamamatsu University School of Medicine, Handayama 1-20-1, Hamamatsu 431-3192, Japan

**Keywords:** glioblastoma, glioma, survival, chemosensitivity test, apoptosis

## Abstract

We retrospectively investigated the efficacy and feasibility of individualised chemotherapy based on *in vitro* drug sensitivity testing (DST) for patients with glioblastoma multiforme. A total of 40 consecutive patients with glioblastoma multiforme (GM) were enrolled into this study between January 1995 and December 2000. The flow cytometric (FCM) detection of apoptosis was used to determine the *in vitro* sensitivity of tumour cells obtained at surgery to 30 different kinds of anticancer agents. From the results of FCM assay, an *in vitro* best regimen was prospectively selected. All the patients concurrently received the individualised chemotherapy with the *in vitro* best regimen and 60 Gy of conventional radiation therapy. Of the 31 assessable patients, eight patients (26%) achieved partial response, and 20 patients (65%) had stable disease. The median survival time was 20.5 months. The individualised chemotherapy based on *in vitro* DST was associated with favourable survival time for the patients with GM compared with the reported results of conventional therapy regimens. The present result suggests that the currently available anticancer agents could be effective against GM when used in individualised chemotherapy.

Glioblastoma multiforme (GM) is one of the most refractory cancers to chemotherapy. The best treatment currently available consists of cytoreductive surgery, followed by 60 Gy of radiation therapy and chemotherapy using nitrosoureas that can penetrate the blood–brain barrier (BBB) (Shapiro, 1999). This conventional treatment regimen results in approximately 1 year of median survival for GM and 2 years for anaplastic astrocytoma (AA) in consecutive, nonselected patients populations (Takakura *et al*, 1986; Levin *et al*, 1990; Hildebrand *et al*, 1994; Iwadate *et al*, 1995; Medical Research Council Brain Tumour Working Party, 2001). Although chemotherapy contributes to modest but significant improvement in the survival time of AA, no study has demonstrated that chemotherapy was linked with an improvement in the survival of GM patients (Levin *et al*, 1990; Hildebrand *et al*, 1994).

Present cancer treatment requires the selection of uniform therapy regimen for patient disease categories on the basis of clinical trials in large populations of the patients. Genetic alterations and gene expression patterns, however, are heterogeneous even in the same histological type of tumours, which leads to heterogeneous responses to the cancer therapies (James and Olson, 1995; Iwadate *et al*, 1996,^1998^,^2000^; Rickman *et al*, 2001). This heterogeneity in drug sensitivity would partly account for the relative lack of success with uniform and conventional chemotherapy regimens obtained by such a clinical trial (Shapiro, 1999). The chemotherapy with optimised combinations of anticancer agents according to the heterogeneity in chemosensitivity could improve the survival of the patients.

Many *in vitro* drug-sensitivity tests (DSTs) have been examined for their predictive accuracy (Salmon *et al*, 1978; Mosmann, 1983; Rosenblum *et al*, 1983; Kimmel *et al*, 1987, Iwadate *et al*, 2002). Among the various assay systems, simple and reliable methods would enable the design of a specific regimen for an individual patient in routine clinical works, sparing patients with resistant tumours the toxicity of ineffective treatment. We have shown that the flow cytometric (FCM) analysis of DNA integrity, which detects apoptosis quantitatively as a population of sub-G_1_ peak, is feasible and sufficiently reliable as a routine clinical DST (Iwadate *et al*, 1997). The clinical sensitivity was predicted in 86% of all evaluable patients and clinical resistance in 81%; the overall accuracy of the FCM assay was 82% for intracerebral gliomas (Iwadate *et al*, 2002).

There are few prospective trials on chemotherapy regimens selected by *in vitro* DST for extracerebral cancers (Alonso, 1984; Gazdar *et al*, 1990, Von Hoff *et al*, 1990, Sekiya *et al*, 1991; Cortazar *et al*, 1997), and no prospective trial for malignant gliomas. To verify the hypothesis that chemotherapy with optimised combinations of anticancer agents could improve the survival of patients with GM, we performed a clinical trial of chemotherapy with agents prospectively selected by the FCM assay for each patient (‘individualised chemotherapy’).

## MATERIALS AND METHODS

### Eligibility

This was a multi-institutional study conducted at Chiba University Hospital and Chiba Cancer Center Hospital to test the hypothesis that optimised combinations of anticancer agents prospectively selected by DST could improve the survival of patients with GM. The primary end point of the study was the overall survival time of patients who were treated with anticancer agents selected by DST. Patients were included in the trial if they had been histologically confirmed, newly diagnosed supratentorial GM. The pathology specimens obtained at surgical excision or biopsy were reviewed by a neuropathologist. Other eligibility criteria included the following; (1) age of 15 years or older; (2) Karnofsky performance status (KPS) of 50% or greater at the time of assignment; (3) adequate organ function defined by WBC ⩾3000 *μ*L^−1^, platelets⩾100 000 *μ*L^−1^, haemoglobin⩾10.0g dL^−1^, bilirubin less than 1.5 mg dL^−1^, AST less than two times the upper limit of normal, and creatinine less than 2.0 mg dL^−1^. The patients were informed of the investigational nature of the study and were required to provide informed consent.

Pretreatment evaluation included a complete medical history, a physical examination, and a detailed neurological examination. Laboratory tests included a complete blood count with a platelet count and blood chemistry. Computed tomography (CT) with contrast enhancement and magnetic resonance imaging (MRI) scan with and without gadolinium was performed preoperatively and postoperatively before the initiation of radiotherapy and chemotherapy.

### *In vitro* DST

We used direct *in vitro* measurement of apoptosis as DST. Two different methods were employed as described previously; FCM analysis of DNA integrity, by which apoptosis is represented as the sub-G_1_ population (Iwadate *et al*, 1997,^2002^), and morphological observation of nuclear damages (Sekiya *et al*, 1991). Surgically resected tumour cells were immediately minced and suspended in RPMI 1640 supplemented with 10% fetal calf serum. The micro-cell aggregate suspension was passed through a sterilised mesh several times to remove fibrous connective tissue, and centrifuged at 1000 r.p.m. for 5 min to eliminate fatty tissues and necrotic portions. An aliquot of the cell suspension was incubated individually with 30 different kinds of anticancer agents (Table 1

**Table 1:** Anticancer agents: *in vitro* concentrations and *in vivo* administration doses

**Drugs**	**Abbreviations**	***In vitro***	***In vivo***
*Alkylating agents*			
Cyclophosphamide	CPM	0.1 *μ*g ml^−1^, 1 *μ*g ml^−1^	750 mg m^−2^
Ifosphamide	IFOS	0.1 *μ*g ml^−1^, 1 *μ*g ml^−1^	1.0 g m^−2^ × 5d
Melphalan	MPL	0.5 *μ*g ml^−1^	NU
Carboquone	CQ	0.1 *μ*g ml^−1^	NU
Nimustine	ACNU	2 *μ*g ml^−1^, 20 *μ*g ml^−1^	75 mg m^−2^
Ranimustine	MCNU	2 *μ*g ml^−1^	75 mg m^−2^
Cisplatin	CDDP	0.5 *μ*g ml^−1^, 5 *μ*g ml^−1^	20 mg m^−2^ × 5d
Carboplatin	JM-8	4 *μ*g ml^−1^	400 mg m^−2^
			
*Topoisomerase inhibitors*			
Actinomycin D	ACD	0.01 *μ*g ml^−1^	NU
Adriamycin	ADM	0.3 *μ*g ml^−1^, 3 *μ*g ml^−1^	40 mg m^−2^
Daunomycin	DM	0.6 *μ*g ml^−1^	40 mg m^−2^ × 2d
Pirarubicin	THP	0.3 *μ*g ml^−1^	40 mg m^−2^
Epirubicin	4′-EPI	0.4 *μ*g ml^−1^	40 mg m^−2^
Aclarubicin	ACR	0.3 *μ*g ml^−1^, 0.6 *μ*g/ml	40 mg m^−2^
Mitoxantrone	MIT	0.06 *μ*g ml^−1^	12 mg m^−2^ × 3d
Etoposide	VP-16	3 *μ*g ml^−1^, 30 *μ*g ml^−1^	60 mg m^−2^ × 5d
Camptothecin	CPT-11	3 *μ*g ml^−1^	NU
			
*Antimetabolites*			
Methotrexate	MTX	3 *μ*g ml^−1^	40 mg m^2^ × 2d
5-Fluorouracil	5-FU	10 *μ*g ml^−1^	800 mg d^−1^ (p.o.)
Thioinosine	6-MPR	3 *μ*g ml^−1^	2 mg kg^−1^ (p.o.)
Cytosine arabinoside	CA	4 *μ*g ml^−1^, 40 *μ*g ml^−1^	60 mg m^−2^ × 7d
			
*Antibiotics*			
Mitomycin C	MMC	0.2 *μ*g ml^−1^, 2 *μ*g ml^−1^	10 mg m^−2^
Bleomycin	BLM	1 *μ*g ml^−1^	10 U m^−2^
Peplomycin	PEP	0.1 *μ*g ml^−1^, 0.5 *μ*g ml^−1^	NU
Chlomomycin A3	TM	0.01 *μ*g ml^−1^	NU
Neocarzinostatin	NCS	0.15 *μ*g ml^−1^	NU
			
*Antimicrotubule agents*			
Vincristine	VCR	0.02 *μ*g ml^−1^, 0.1 *μ*g ml^−1^	1 mg m^−2^
Vinblastine	VLB	0.1 *μ*g ml^−1^	5 mg m^−2^
Vindesine	VDS	0.1 *μ*g ml^−1^	3 mg m^−2^
Paclitaxel	TAX	0.6 *μ*g ml^−1^, 6 *μ*g ml^−1^	100 mg m^−2^

NU=not used in this study.

). *In vitro* drug concentrations were set at 1/10 of the peak plasma concentration when used in the clinically recommended doses (Alberts and Chen, 1980), and the dose–response evaluation was performed in selected 12 drugs. To evaluate the pro-drugs adequately, the *in vivo* activated forms, 4-hydroperoxycyclophosphamide and 4-hydroperoxyifosphamide, were used for cyclophosphamide and ifosfamide, respectively. Cells were incubated with each agent at 37°C in 5% CO_2_ for 8 h, and then cultured in fresh drug-free RPMI 1640 medium with 10% fetal calf serum for 72 h. Then, the FCM analysis of propidium iodide (PI)-stained nuclei was performed. The treated cells were mixed with phosphate buffered-saline pH 7.2/0.1% Triton X-100/0.1 mg ml^−1^ RNase/0.01% sodium azide for 15 min, and then with 50 *μ*g ml^−1^ propidium iodide (Sigma, St Louis, MO, USA) for 5 min. Isolated nuclei were analysed with a flow cytometer (FACScan: Becton Dickinson, Mountain View, CA, USA). As apoptotic nuclei shifted to the hypodiploid (sub-G_1_) area, the integrated diploid peak (G_0_/G_1_ peak) reciprocally decreased. The effectiveness of the drugs was judged by reduction in the G_0_/G_1_ peak compared with that of untreated control cells; more than 20% reduction was judged as positive. In addition to the FCM assay, drug-induced apoptosis was confirmed morphologically. The treated cells were fixed with 70% ethanol and stained with Giemsa on a slide glass. A total of 400 nuclei per slide were observed using the high-power field of a light microscope. Apoptotic changes noted in the nuclei, such as chromatin degradation or condensation, were judged as markers of apoptosis, and counted to compare with nontreated controls. The Fisher’s exact probability test was used to examine the statistical difference under the level of 5% between the samples and the nontreated control. This morphological study showed that the average percentages of tumour cells in the preparations were constantly over 95. The DNA integrity assessed by the FCM analysis was well correlated with the morphological changes in the nuclei. Drugs were finally determined as effective *in vitro* when either of the assays was judged as positive to avoid a false-negative result.

### Treatment protocol

Two or three anticancer agents were prospectively selected for each patient from the results of the DST. When a number of agents were effective against the tumour sample *in vitro*, the agents showing the highest grade of effectiveness were chosen. In the case where several agents showed almost the same grade of effectiveness, we chose agents based on their ability to penetrate the BBB, and preferred to the combination of drugs with different mechanism of pharmaceutical action. When no agent was positive *in vitro*, the patients were treated only with radiation therapy. These patients were included in the survival study so as to avoid an exclusion bias. The doses and schedules of individualised chemotherapy regimens were determined on the basis of the clinically recommended doses (Table 1). All regimens were given at least every 3 months for a year. The schedules of individualised chemotherapy were modified from previously published regimens to produce similar haematological toxicity. Conventional radiation therapy with a megavoltage machine was begun within 2 weeks after surgical removal in conjunction with the chemotherapy. The initial treatment volume included the contrast-enhancing lesion surrounding edema plus a 2-cm margin by the preoperative CT and MRI scan. This treatment volume received a dose of 40 Gy in 2-Gy fractions, and an additional 20 Gy in 2-Gy fractions was delivered to the boost volume, which included the contrast-enhancing lesion plus a 5-mm margin.

### Evaluation methods

This trial was designed to estimate the survival time of GM patients treated with individualised chemotherapy combined with conventional radiotherapy. Survival duration was calculated from the date of surgery until the date of last follow-up or death. Survival curves were generated using the Kaplan–Meier method. To evaluate the initial tumour response to the individualised chemotherapy, MRI studies were performed before the patient entered the study, after induction chemotherapy, and after every cycle of maintenance chemotherapy. Responses were based primarily on the product of the largest two tumour diameters seen on the MRI study, and were evaluated according to the response evaluation criteria in solid tumours (RECIST) (Therasse *et al*, 2000). Complete response (CR) was defined as the disappearance of all measurable enhancing tumour on imaging studies for more than 4 weeks, and neurologically stable or improved. Partial response (PR) was defined as a reduction of 50% or greater in the sum of the products of the largest perpendicular diameters of contrast enhancement for all measurable lesions or definitely better for all nonmeasurable lesions on MRI scans for more than 4 weeks. No new lesions could arise. Progressive disease (PD) was defined as a 25% or greater increase in the size of the products of the largest perpendicular diameters of contrast enhancement for any measurable lesions or definitely worse for all nonmeasurable lesions or any new tumour on MRI scans. Stable disease (SD) was defined as all other situations.

## RESULTS

### Patient characteristics

Consecutive 40 patients who were referred to the two institutes between January 1995 and December 2000, were enrolled in this study. Patient age, sex, surgical intervention, KPS score, and tumour location are listed in Table 2

**Table 2:** Patient characteristics

No. of cases	40
*Age (years)*	
Mean	51
Range	18∼70
	
*Sex*	
Male	24
Female	16
	
*KPS score*	
Mean	76
Range	50∼100
	
*Tumour location*	
Left hemisphere	21 (53%)
Right hemisphere	13 (33%)
Midline	6 (15%)
	
*Extent of surgery*	
Biopsy	4 (10%)
Partial	11 (28%)
Subtotal	19 (48%)
Total	6 (15%)

KPS=Karnofsky performance status.

. Patients had a mean age of 51 years (range, 18–70 years). In all, 45% of the patients with GM were less than 50 years of age. The mean KPS score was 76 at the time of diagnosis (range, 50–100). All patients underwent maximal tumour resection with stereotactic biopsy reserved only for unresectable tumours. In all, 19 patients (48%) underwent a subtotal resection of the tumour, and six patients (15%) had a total resection.

### *In vitro* effective agents against GM

Specimens from the 40 patients with GM were investigated by means of the FCM analysis of DNA integrity and morphological changes of apoptosis for their susceptibility to the 30 anticancer agents that are in clinical use. In this series of newly diagnosed cases with GM, the successful rate of *in vitro* assay was 100%. The results showed that effective agents were markedly heterogeneous among the patients. Three specimens were judged as negative for all the 30 anticancer agents. The patients without *in vitro* effective agents were treated with radiotherapy alone, and were excluded from the initial response evaluation but included in the survival analysis. *In vitro* effective rates were calculated for each drug (Table 3

**Table 3:** *In vitro* effective rates for each anticancer agent

**Drugs**	**No. of effective cases**	**Effective rate (%)**
*Alkylating agents*		
Cyclophosphamide	4	10
Ifosphamide	4	10
Melphalan	1	2.5
Carboquone	2	5
Nimustine	1	2.5
Ranimustine	4	10
Cisplatin	9	18
Carboplatin	2	5
		
*Topoisomerase inhibitors*		
Actinomycin D	0	0
Adriamycin	3	7.5
Daunomycin	6	15
Pirarubicin	5	12.5
Epirubicin	3	7.5
Aclarubicin	10	25
Mitoxantrone	3	7.5
Etoposide	8	20
Camptothecin	1	2.5
		
*Antimetabolites*		
Methotrexate	2	5
5-Fluorouracil	3	7.5
Thioinosine	2	5
Cytosine arabinoside	5	12.5
		
*Antibiotics*		
Mitomycin C	4	10
Bleomycin	2	5
Peplomycin	2	5
Chlomomycin A3	1	2.5
Neocarzinostatin	1	2.5
		
*Antimicrotubule agents*		
Vincristine	6	15
Vinblastine	3	7.5
Vindesine	5	12.5
Paclitaxel	6	15

), and were relatively high in aclarubicin (25%), etoposide (20%), and cisplatin (18%). The present result showed that the effective rates of nitrosoureas, which are the gold standard for the chemotherapy against GM, was not so high; effective only in one case (2.5%) for ACNU and in four cases (10%) for MCNU.

### Response to treatment

The best initial response to the treatment is summarised in Table 4

**Table 4:** Initial tumour response to the individualised chemotherapy

	**CR**	**PR**	**SD**	**PD**	**NE**	***n***	**RR**
Glioblastoma multiforme	0	8	20	3	9	40	26%

CR=complete response; PR=partial response; SD=stable disease; PD=progressive disease; NE=not evaluable; RR=response rate.

. Nine patients were not assessable: six patients had no residual tumours after surgery and three patients did not receive chemotherapy because of the all-negative results in the *in vitro* DST. Of the 31 assessable patients, there were no CR and eight PRs (objective response rate, 26%; 95% confidence interval). In total, 20 patients (65%) achieved SD lasting more than 3 months.

### Overall survival

**Figure 1:**
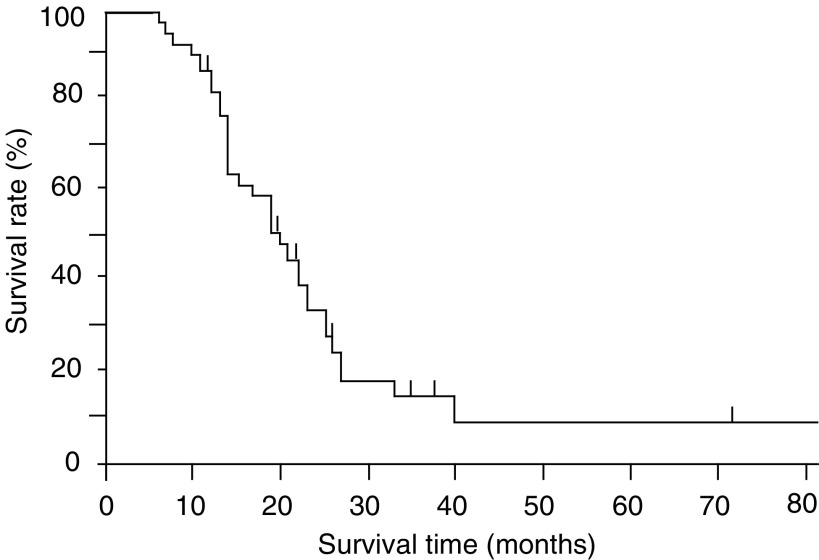
Kaplan–Meier overall survival for the patients with GM.

The survival time, measured from the time of the first resection or biopsy, was analysed based on the Kaplan–Meier product-limit method (Figure 1

). The median survival time was 20.5 months, and the long-term survival rate over 3 years was approximately 10%.

## DISCUSSION

Recent advance in the molecular biological analysis of gliomas has revealed that histologically-identical tumours possess heterogeneous gene alterations and thus exhibit heterogeneous sensitivity to anticancer agents (James and Olson, 1995; Iwadate *et al*, 1996,^1998^,^2000^). *In vitro* DST would provide a rationale for the selection of therapy for individual patient on the basis of biological characteristics of the patient’s tumour (Kimmel *et al*, 1987). However, it has not been elucidated whether chemotherapy with prospectively selected agents can improve the patients’ survival compared with conventional and uniform therapy regimens. We demonstrated, in the present study, that the chemotherapy with agents prospectively selected by DST for each patient (individualised chemotherapy) was associated with favourable survival time in patients with GM. Indeed, the median survival of 20.5 months in consecutive and nonselected GM patients compares favourably with most other reported series. This is the first report to show that the individualised chemotherapy selected by *in vitro* DST may contribute to prolongation of the survival period of GM patients.

The methods to predict the clinical response of individual patients to chemotherapy are classified roughly into two classes. One approach is molecular tumour analyses including the gene expression profiles obtained with DNA microarray technology, which can in part predict the cellular response to the anticancer agents from the gene expression profile (Dan *et al*, 2002). The other approach is the *in vitro* DST using cell-culture technique (Kimmel *et al*, 1987). The DST frequently used at this time is the colony-forming assay (CFA) and the 3-(4,5-dimethylthiazol-2-yl)-2,5-diphenyltetrazolium bromide (MTT) assay (Salmon *et al*, 1978; Mosmann, 1983; Rosenblum *et al*, 1983). Although the CFA is considered to be reliable for predicting the clinical response to therapies, it is time- and cost-consuming, labour-intensive, and suffers from a low success rate. It is because few prospective trials for gliomas have attempted to select prospective therapy on the basis of *in vitro* analyses. In this study, we used a direct *in vitro* measurement of apoptosis by the FCM analysis of DNA integrity as a DST. This method has been widely used in basic laboratory studies to detect apoptosis quantitatively (Nicoletti *et al*, 1991; Darzynkiewicz *et al*, 1992; Iwadate *et al*, 1997). When used as a DST, the clinical sensitivity was predicted in 86% and clinical resistance in 81% for the patients with intracerebral gliomas (Iwadate *et al*, 2002). Since the flow cytometer directly measure the nuclear damage of each tumour cell at the final stage of the assay, strict single-cell suspension is theoretically unnecessary at the initial stage. This feature would contribute to the high success rate and the high predictive accuracy. Although the *in vitro* DST suffers from difficulties in duplicating the complex conditions of *in vivo* therapy, they would have some advantages over the gene expression analyses because numerous known and unknown molecular networks influence the susceptibility of the tumour cells to anticancer agents.

The initial response rate for GM was 26% in the present series, and the SD was accomplished in 65% of the patients. These results suggest that the individualised chemotherapy is not curative, but effectively induces the tumour to enter a dormant state. Because the currently available anticancer agents have low tumour-specificity, a complete cure of solid tumours such as GM cannot be obtained with chemotherapy. The main advantage of individualised chemotherapy using an optimised combination of agents seems to be that tumour dormancy can be obtained without dose-escalation. In summary, it is reasonable to conclude from the present study that chemotherapy even with the currently available agents provides an opportunity to improve the survival of patients with GM when applied in a specific regimen prospectively selected for individual patients using *in vitro* DST. However, further observations will be required to determine whether the potential therapeutic benefit is directly associated with administering the individualised chemotherapy. The present result warrants the use of this strategy in a larger controlled randomised study.
